# Meta-analysis of efficacy and adverse events of erlotinib-based targeted therapies for advanced/metastatic non-small cell lung cancer

**DOI:** 10.18632/oncotarget.19735

**Published:** 2017-07-31

**Authors:** Fei Li, Shu-Hua Zhang, Li-Min Pang

**Affiliations:** ^1^ Department of Radiotherapy, China-Japan Union Hospital of Jilin University, Changchun 130033, China; ^2^ Department of Operation, China-Japan Union Hospital of Jilin University, Changchun 130033, China; ^3^ Department of Pediatrics, China-Japan Union Hospital of Jilin University, Changchun 130033, China

**Keywords:** advanced/metastatic non-small-cell lung cancer, targeted therapy, efficacy, adverse events, randomized controlled trials

## Abstract

A network meta-analysis evaluating efficacy and adverse events of eight erlotinib-based therapies (erlotinib+placebo, erlotinib+tivantinib, erlotinib+celecoxib, erlotinib+onartuzumab, erlotinib+sunitinib, erlotinib+entinostat, erlotinib+sorafenib, and erlotinib+bevacizumab) for advanced/metastatic non-small-cell lung cancer (NSCLC) was performed. PubMed and Cochrane Library were reviewed, and ten randomized controlled trials were identified in which patients receiving at least one erlotinib-based therapy. Efficacy outcomes, including progression-free survival (PFS), overall survival (OS), overall response rate (ORR), disease control rate (DCR), and adverse outcomes were evaluated. Patients treated with erlotinib+tivantinib, or erlotinib+celecoxib had longer PFS than patients on erlotinib+placebo; patients on erlotinib+tivantinib had longer OS compared to erlotinib+placebo. For PFS, erlotinib+celecoxib had the highest value of surface under the cumulative ranking curve (SUCRA). For OS, erlotinib+tivantinib had the highest SUCRA. For ORR, erlotinib+bevacizumab had the highest SUCRA, while erlotinib+entinostat ranked the lowest. For DCR, erlotinib+sorafenib had the highest SUCRA. Erlotinib+onartuzumab had the highest SUCRA for diarrhea, nausea, vomiting, decreased appetite, and dyspnea. Erlotinib+sunitinib had the lowest SUCRA for diarrhea, nausea, vomiting, and decreased appetite. Erlotinib + entinostat had the lowest SUCRA for fatigue, asthenia, and dyspnea. Our study suggests erlotinib+tivantinib and erlotinib+celecoxib regimens have the best long-term efficacy, while erlotinib+sunitinib and erlotinib+entinostat have the fewest adverse effects in patients with advanced/metastatic NSCLC.

## INTRODUCTION

Non-small cell lung cancer (NSCLC) is the most common type of lung cancer, and often develops into an advanced or metastatic disease [1]. Internal genetic and environmental factors, such as smoking, radon, and asbestos have been reported as possible etiologic factors of NSCLC [2, 3]. Currently, platinum-based chemotherapy and the application of epidermal growth factor receptor (EGFR) inhibitor erlotinib have been the main strategies for the treatment of advanced/metastatic NSCLC [4]. In NSCLC, c-MET (MET) receptor tyrosine kinase has been associated with the development of resistance to EGFR tyrosine kinase inhibitors (TKIs) in various EGFR-mutant cancers [5, 6]. Combined inhibition of VEGFR and EGFR therapy has been used in patients with advanced pretreated NSCLC, using sunitinib plus erlotinib [7].

Erlotinib, a small molecule inhibitor of EGFR, has been used for the treatment of advanced/metastatic NSCLC patients who do not respond to chemotherapy regimens [1, 8, 9]. Moreover, erlotinib can improve survival in patients with untreated NSCLC who have EGFR-activating mutations [10]. Combination treatments for advanced/metastatic NSCLC are becoming more popular, since they may be more effective than a single drug treatment. Combining erlotinib with sorafenib results in a dual inhibition of EGFR signaling and angiogenesis, which are two vital targets in treatment of NSCLC [11]. However, a combined therapy using entinostat and erlotinib is still controversial, since patients with advanced NSCLC do not respond to the treatment [12]. Several studies have evaluated the efficacy of erlotinib-based targeted therapies, but with mixed results [4, 7, 13, 14]. In addition, agents like erlotinib and bevacizumab may cause inevitable adverse events including rash, diarrhea, dry skin, and fatigue; therefore, it is necessary to determine which targeted therapy is safer and produces less toxicity [15].

A meta-analysis integrates the results of various independent studies, thus increasing the statistical power [16]. In this study, we employed a network meta-analysis approach to compare the efficacy and adverse events among eight targeted, erlotinib-based therapies (regimens of erlotinib + placebo, erlotinib + tivantinib, erlotinib + celecoxib, erlotinib + onartuzumab, erlotinib + sunitinib, erlotinib + entinostat, erlotinib + sorafenib and erlotinib + bevacizumab) for patients with advanced/metastatic NSCLC.

## RESULTS

### Baseline characteristics of eligible studies

A total of 1,991 published studies were initially identified through electronic databases and manual searches. After excluding duplicates (*n* = 9), letters, reviews or abstracts (*n* = 260), non-human studies (*n* = 199) and non-English studies (*n* = 245), the remaining 1278 studies were examined. Subsequently, non-randomized control trials (*n* = 367), studies not relevant to advanced/metastatic NSCLC (*n* = 306), studies not relevant to targeted therapies (*n* = 594) and a study with uncomplete data (*n* = 1) were also excluded. Finally, 10 randomized controlled trials that met inclusion criteria were enrolled in this meta-analysis [1, 4–7, 10, 11, 15, 17] (Supplementary Figure 1). There were 3,792 patients with advanced/metastatic NSCLC; patients who received erlotinib + placebo, and erlotinib + tivantinib regimens accounted for the majority. All eligible studies were published between 2011 and 2015. One study included Asian subjects while nine studies included European and American subjects. All 10 eligible studies were two-arm trials. Baseline characteristics of eligible studies and the Cochrane risk of bias assessment are presented in Supplementary Table 1 and Supplementary Figure 2.

### Pairwise meta-analysis

We conducted a pairwise meta-analysis to compare the efficacy and adverse events of eight targeted therapies for patients with advanced/metastatic NSCLC. The results indicated that in terms of efficacy and PFS (months), erlotinib + placebo regimen had a shorter PFS when compared with erlotinib + tivantinib, and erlotinib + sunitinib regimens (WMD = −1.64, 95% CI = −1.82 ∼ -1.45; WMD = −1.21, 95% CI = −1.99 ∼ −0.42, respectively), suggesting that the efficacies of erlotinib + tivantinib, and erlotinib + sunitinib regimens were better than that of erlotinib + placebo regimen. In terms of OS (months), the OS of patients taking erlotinib + placebo regimen was shorter than the OS of patients taking erlotinib + tivantinib, and erlotinib + sunitinib regimens (WMD = −1.83, 95% CI = −2.08 ∼ −0.57; WMD = −0.60, 95% CI = −0.58 ∼ −0.42, respectively), indicating that erlotinib + tivantinib, and erlotinib + sunitinib regimens had better efficacy for patients with advanced/metastatic NSCLC. In terms of ORR, patients taking erlotinib + placebo regimen had relatively lower ORR than patients taking erlotinib + sunitinib regimen (OR = 0.62, 95% CI = 0.40 ∼ 0.97), indicating that erlotinib + sunitinib regimen had better efficacies than erlotinib + placebo regimen. In terms of DCR, a variety of targeted therapies showed no significant difference in the patients with advanced/metastatic NSCLC (Table 1).

**Table 1:** Weighted mean difference or odds ratio (95% CI) of pairwise meta-analysis

Studies	Comparison	Pairwise meta-analysis		
		WMD/OR (95% CI)	*I*^2^	*P*_h_
**Efficacy**				
**PFS (months)**				
2 studies	A vs B	**−1.64 (−1.82∼–1.45)**	63.3%	0.099
2 studies	A vs E	**−1.21 (−1.99∼–0.42)**	97.7%	< 0.0001
**OS (months)**				
3 studies	A vs B	**−1.83 (−2.08∼–0.57)**	96.6%	< 0.0001
2 studies	A vs E	**−0.60 (−0.58∼–0.42)**	0.0%	0.809
**ORR**				
2 studies	A vs E	**0.62 (0.40∼0.97)**	0.0%	0.980

Notes: PFS and OS are stated as WMD (95% CI), while ORR and DCR are presented as OR (95%CI). 95% CI = 95% confidence interval; WMD = Weighted mean difference; OR = Odds ratio; PFS = progression-free survival; OS = overall survival; ORR = overall response rate; DCR = disease control rate; A = Erlotinib + Placebo; B = Erlotinib + Tivantinib; E = Erlotinib + Sunitinib.

In terms of adverse events and diarrhea, the rate of diarrhea in patients taking erlotinib + placebo regimen was higher than in patients taking erlotinib + tivantinib regimen (OR = 1.31, 95%CI = 1.04∼1.65). In contrast, compared with patients taking erlotinib + sunitinib regimen, patients taking erlotinib + placebo regimen had a lower rate of diarrhea (OR = 0.28, 95% CI = 0.16∼0.46). Compared with patients taking erlotinib + sunitinib regimen, patients taking erlotinib + placebo regimen had lower incidences of fatigue or asthenia, nausea or vomiting and decreased appetite (OR = 0.67, 95% CI = 0.50∼0.89; OR = 0.52, 95% CI = 0.39∼0.68; OR = 0.46, 95% CI = 0.34∼0.62, respectively). In terms of dyspnea, four targeted therapies (regimens of erlotinib + placebo, erlotinib + tivantinib, erlotinib + onartuzumab and erlotinib + entinostat) exhibited no significant difference in dyspnea in patients with advanced/metastatic NSCLC (Table 2).

**Table 2:** Estimated OR and 95% CI produced by random effects pairwise meta-analysis for adverse events in advanced/metastatic non-small-cell lung cancer (NSCLC) patients

Included studies	Comparisons	Adverse events		Pairwise meta-analysis		
		Treatment1	Treatment2	OR (95% CI)	*I*^2^	*P*_h_
**Adverse events (all grades)**						
**Diarrhea**						
2 studies	A vs. B	257/600	220/604	**1.31 (1.04∼1.65)**	0.00%	0.981
2 studies	A vs. E	189/541	368/537	**0.28 (0.16∼0.46)**	53.00%	0.145
**Fatigue or Asthenia**						
2 studies	A vs. B	228/600	254/604	0.87 (0.63∼1.21)	23.50%	0.253
2 studies	A vs. E	103/541	140/537	**0.67 (0.50∼0.89)**	0.00%	0.794
**Nausea or Vomiting**						
2 studies	A vs. B	239/600	238/604	0.92 (0.58∼1.47)	55.00%	0.136
2 studies	A vs. E	120/541	191/537	**0.52 (0.39∼0.68)**	0.00%	0.604
**Decreased appetite**						
2 studies	A vs. E	86/541	156/537	**0.46 (0.34∼0.62)**	0.00%	0.48
**Dyspnoea**						
2 studies	A vs. B	139/600	154/604	0.87 (0.67∼1.14)	6.40%	0.301

Notes: OR = odds ratio; 95% CI = 95% confidence interval; NA = not available; A = Erlotinib + Placebo; B = Erlotinib + Tivantinib; E = Erlotinib + Sunitinib.

### Network relationship evidence

**Figure 1:**
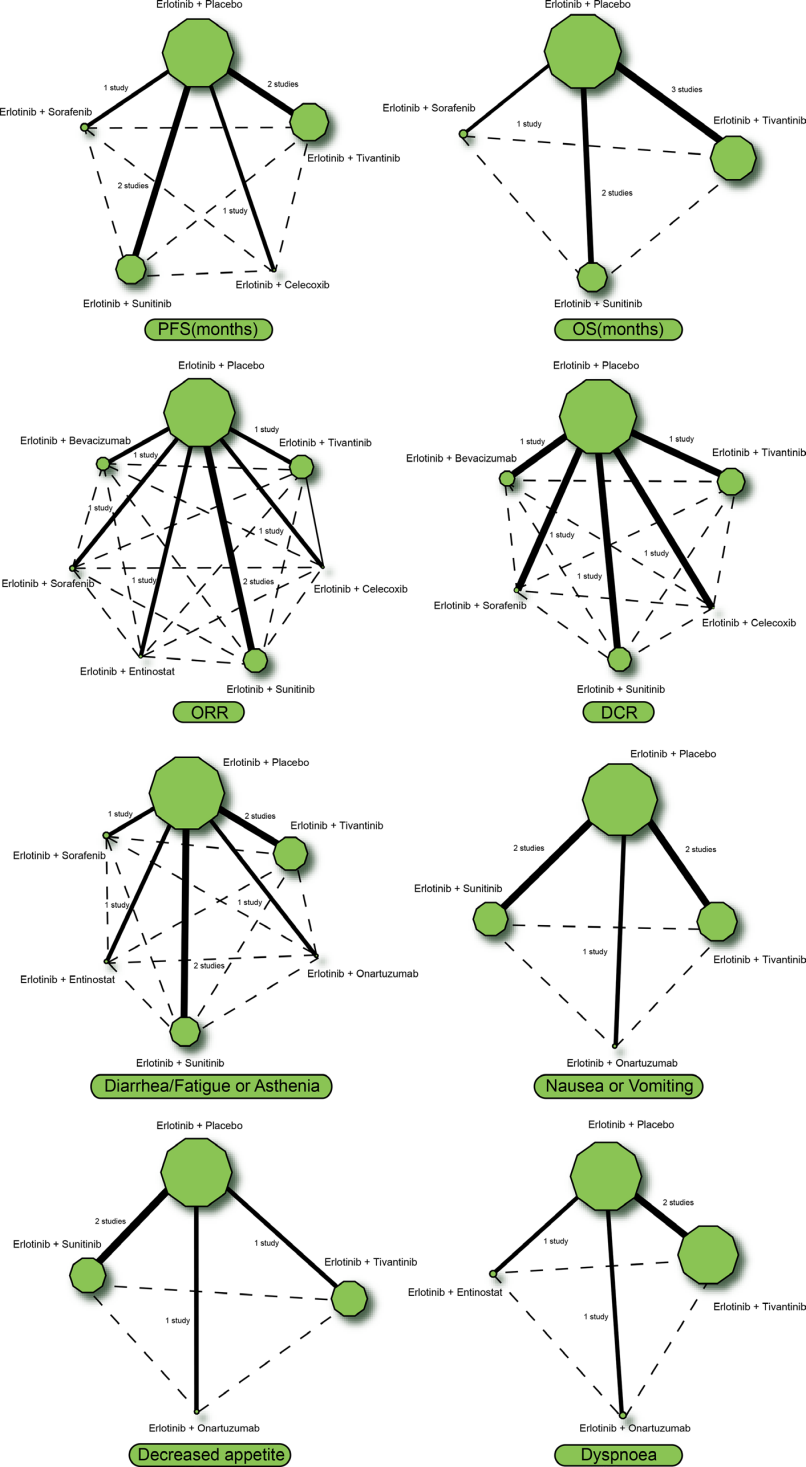
Network evidence diagrams of PFS, OS, ORR, DCR, diarrhea/fatigue or asthenia, nausea or vomiting, decreased appetite and dyspnoea Note: PFS = progression-free survival; OS = overall survival; ORR = overall response rate; DCR = disease control rate.

This study consisted of eight targeted therapies, including regimens of erlotinib + placebo, erlotinib + tivantinib, erlotinib + celecoxib, erlotinib + onartuzumab, erlotinib + sunitinib, erlotinib + entinostat, erlotinib + sorafenib and erlotinib + bevacizumab. It was observed that more patients with advanced/metastatic NSCLC took erlotinib + placebo and erlotinib + tivantinib regimens, while fewer patients took erlotinib + celecoxib and erlotinib + entinostat regimens (Figure 1).

### Main results of network meta-analysis

**Figure 2:**
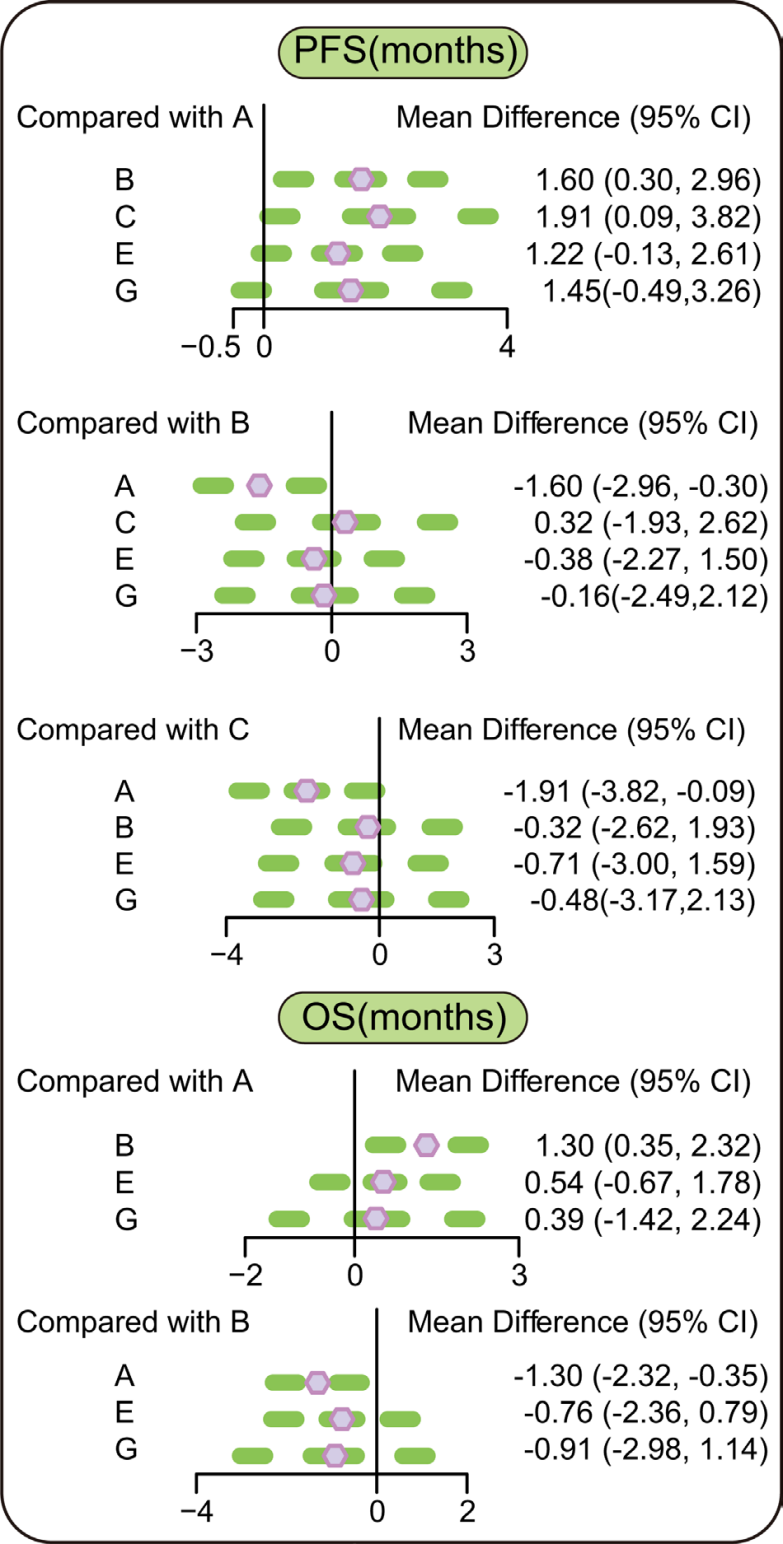
Relative relationship forest plots of PFS and OS Note: PFS = Progression-free survival; OS = overall survival; 95% CI = 95% confidence interval; A = erlotinib + placebo regimen; B = erlotinib + tivantinib regimen; C = erlotinib + celecoxib regimen; E = erlotinib + sunitinib regimen; G = erlotinib + sorafenib regimen.

The results of network meta-analysis indicated that in terms of efficacy and PFS (months), patients with advanced/metastatic NSCLC who took erlotinib + tivantinib and erlotinib + celecoxib regimens had a relatively longer PFS compared with patients who took erlotinib + placebo regimen (WMD = 1.60, 95% CI = 0.30∼2.96; WMD = 1.91, 95% CI = 0.09∼3.82, respectively), suggesting that the efficacy of erlotinib + tivantinib and erlotinib + celecoxib regimens was better. In terms of OS (months), patients who were treated with erlotinib + tivantinib regimen had a longer OS than those taking erlotinib + placebo regimen (WMD = 1.30, 95% CI = 0.35∼2.32), indicating that the efficacy of erlotinib + tivantinib regimen was better. In terms of ORR and DCR, there was no significant difference between 7 targeted therapies (Figure 2 and Table 3).

**Table 3:** WMD or OR (95%CI) of seven treatment modalities of four endpoints

WMD/OR(95%CI)						
**Efficacy**						
**PFS (months)**						
A	**1.60 (0.30, 2.96)**	**1.91 (0.09, 3.82)**	1.22 (−0.13, 2.61)	1.45 (−0.49, 3.26)		
**−1.60 (−2.96, −0.30)**	B	0.32 (−1.93, 2.62)	−0.38 (−2.27, 1.50)	−0.16 (−2.49, 2.12)		
**−1.91 (−3.82, −0.09)**	−0.32 (−2.62, 1.93)	C	−0.71 (−3.00, 1.59)	−0.48 (−3.17, 2.13)		
−1.22 (−2.61, 0.13)	0.38 (−1.50, 2.27)	0.71 (−1.59, 3.00)	E	0.23 (−2.12, 2.50)		
−1.45 (−3.26, 0.49)	0.16 (−2.12, 2.49)	0.48 (−2.13, 3.17)	−0.23 (−2.50, 2.12)	G		
**OS (months)**						
A	**1.30 (0.35, 2.32)**	0.54 (−0.67, 1.78)	0.39 (−1.42, 2.24)			
**−1.30 (−2.32, −0.35)**	B	−0.76 (−2.36, 0.79)	−0.91 (−2.98, 1.14)			
−0.54 (−1.78, 0.67)	0.76 (−0.79, 2.36)	E	−0.14 (−2.29, 2.05)			
−0.39 (−2.24, 1.42)	0.91 (−1.14, 2.98)	0.14 (−2.05, 2.29)	G			
**ORR**						
A	1.64 (0.47, 5.64)	0.60 (0.13, 2.36)	1.61 (0.57, 4.75)	0.26 (0.03, 1.76)	0.70 (0.15, 3.61)	2.18 (0.58, 8.46)
0.61 (0.18, 2.11)	B	0.37 (0.05, 2.26)	0.99 (0.19, 5.29)	0.16 (0.01, 1.59)	0.43 (0.06, 3.24)	1.35 (0.23, 7.85)
1.66 (0.42, 7.92)	2.73 (0.44, 19.02)	C	2.74 (0.47, 16.71)	0.45 (0.03, 4.81)	1.19 (0.15, 10.31)	3.77 (0.56, 27.36)
0.62 (0.21, 1.76)	1.01 (0.19, 5.32)	0.36 (0.06, 2.11)	E	0.16 (0.01, 1.44)	0.43 (0.07, 3.00)	1.34 (0.25, 7.31)
3.85 (0.57, 36.17)	6.36 (0.63, 83.38)	2.22 (0.21, 31.35)	6.24 (0.70, 76.33)	F	2.78 (0.24, 42.89)	8.38 (0.84, 113.02)
1.43 (0.28, 6.61)	2.31 (0.31, 16.33)	0.84 (0.10, 6.62)	2.31 (0.33, 14.25)	0.36 (0.02, 4.20)	G	3.15 (0.39, 22.39)
0.46 (0.12, 1.72)	0.74 (0.13, 4.42)	0.27 (0.04, 1.78)	0.74 (0.14, 4.01)	0.12 (0.01, 1.19)	0.32 (0.04, 2.55)	H
**DCR**						
A	1.79 (0.79, 4.10)	1.31 (0.45, 3.94)	1.41 (0.61, 3.29)	1.94 (0.71, 5.30)	1.62 (0.67, 3.80)	
0.56 (0.24, 1.27)	B	0.73 (0.19, 2.93)	0.79 (0.24, 2.56)	1.09 (0.29, 3.85)	0.90 (0.27, 2.91)	
0.76 (0.25, 2.24)	1.38 (0.34, 5.25)	C	1.09 (0.28, 4.08)	1.48 (0.33, 6.59)	1.23 (0.29, 4.74)	
0.71 (0.30, 1.64)	1.27 (0.39, 4.19)	0.92 (0.24, 3.60)	E	1.38 (0.37, 4.94)	1.15 (0.33, 3.71)	
0.52 (0.19, 1.41)	0.92 (0.26, 3.41)	0.67 (0.15, 3.00)	0.73 (0.20, 2.70)	G	0.83 (0.22, 3.15)	
0.62 (0.26, 1.48)	1.12 (0.34, 3.75)	0.81 (0.21, 3.41)	0.87 (0.27, 3.03)	1.20 (0.32, 4.64)	H	

Notes: WMD = Weighted mean difference; OR = Odds ratio; 95% CI = 95% confidence interval, Weighted mean difference or odds ratio (95% CI) below the treatments should be read from row to column while above the treatments should be read from column to row. PFS and OS are stated as WMD (95% CI), while ORR and DCR are presented as OR (95% CI). PFS = Progression-free survival; OS = overall survival; ORR = overall response rate; DCR = Disease control rate; A = Erlotinib + Placebo; B = Erlotinib + Tivantinib; C = Erlotinib + Celecoxib; E = Erlotinib + Sunitinib; F = Erlotinib + Entinostat; G = Erlotinib + Sorafenib; H = Erlotinib + Bevacizumab.

**Figure 3:**
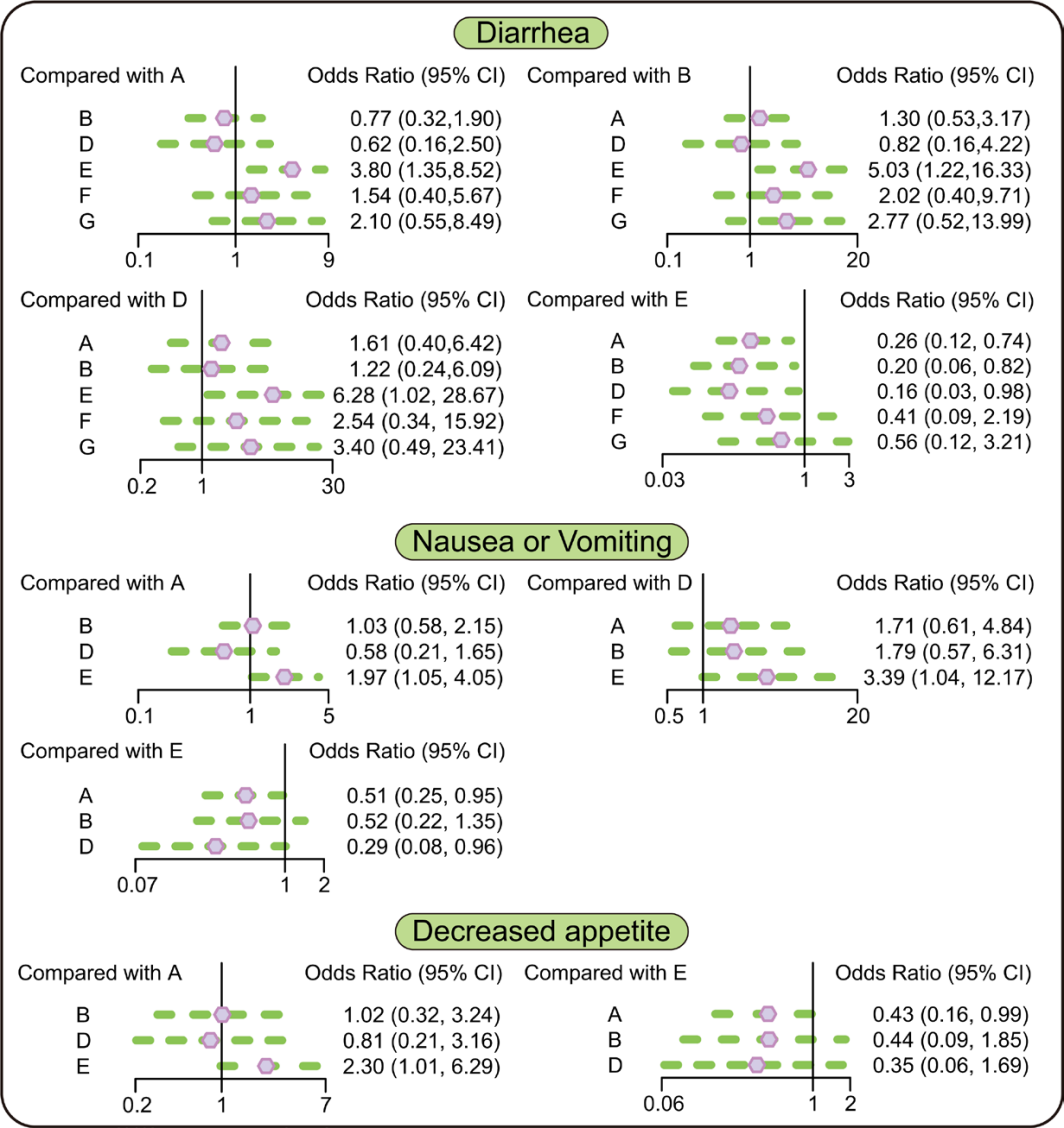
Relative relationship forest plots of diarrhea, nausea or vomiting and decreased appetite Note: A = erlotinib + placebo regimen; B = erlotinib + tivantinib regimen; D = erlotinib + onartuzumab regimen; E = erlotinib + sunitinib regimen; F = erlotinib + entinostat regimen; G = erlotinib + sorafenib regimen.

In terms of adverse events and diarrhea, the rates of diarrhea in patients taking erlotinib + placebo, erlotinib + tivantinib, and erlotinib + onartuzumab regimens were lower than in patients taking erlotinib + sunitinib regimen (OR = 0.26, 95% CI = 0.12∼0.74; OR = 0.20, 95% CI = 0.06∼0.82; OR = 0.16, 95% CI = 0.03∼0.98, respectively). In terms of nausea or vomiting, patients taking erlotinib + placebo, and erlotinib + onartuzumab regimens had less nausea or vomiting compared with patients taking erlotinib + sunitinib regimen (OR = 0.51, 95% CI = 0.25 ∼ 0.95; OR = 0.29, 95%CI = 0.08 ∼ 0.96, respectively). In terms of decreased appetite, compared with patients taking erlotinib + placebo regimen, patients taking erlotinib + sunitinib regimen had a decreased appetite (OR = 2.30, 95% CI = 1.01 ∼ 6.29). In terms of fatigue or asthenia and dyspnea, there was no significant difference between 6 targeted therapies (Figure 3 and Table 4).

**Table 4:** Odds ratio (95% CI) of six treatment modalities of five endpoints

OR (95% CI)					
Adverse events (all grades)					
Diarrhea					
A	0.77 (0.32, 1.90)	0.62 (0.16, 2.50)	**3.80 (1.35, 8.52)**	1.54 (0.40, 5.67)	2.10 (0.55, 8.49)
1.30 (0.53, 3.17)	B	0.82 (0.16, 4.22)	**5.03 (1.22, 16.33)**	2.02 (0.40, 9.71)	2.77 (0.52, 13.99)
1.61 (0.40, 6.42)	1.22 (0.24, 6.09)	D	**6.28 (1.02, 28.67)**	2.54 (0.34, 15.92)	3.40 (0.49, 23.41)
**0.26 (0.12, 0.74)**	**0.20 (0.06, 0.82)**	**0.16 (0.03, 0.98)**	E	0.41 (0.09, 2.19)	0.56 (0.12, 3.21)
0.65 (0.18, 2.49)	0.49 (0.10, 2.50)	0.39 (0.06, 2.94)	2.41 (0.46, 11.37)	F	1.41 (0.21, 9.28)
0.48 (0.12, 1.81)	0.36 (0.07, 1.94)	0.29 (0.04, 2.04)	1.78 (0.31, 8.41)	0.71 (0.11, 4.70)	G
**Fatigue or Asthenia**					
A	1.12 (0.63, 1.85)	1.01 (0.41, 2.59)	1.47 (0.84, 2.58)	1.97 (0.72, 5.17)	1.03 (0.42, 2.48)
0.89 (0.54, 1.58)	B	0.90 (0.33, 2.71)	1.32 (0.63, 2.94)	1.75 (0.58, 5.46)	0.91 (0.33, 2.66)
0.99 (0.39, 2.45)	1.11 (0.37, 3.00)	D	1.46 (0.50, 4.14)	1.95 (0.52, 7.04)	1.00 (0.28, 3.54)
0.68 (0.39, 1.20)	0.76 (0.34, 1.59)	0.69 (0.24, 2.01)	E	1.34 (0.42, 4.06)	0.70 (0.24, 1.99)
0.51 (0.19, 1.39)	0.57 (0.18, 1.72)	0.51 (0.14, 1.93)	0.75 (0.25, 2.38)	F	0.53 (0.14, 2.02)
0.97 (0.40, 2.38)	1.09 (0.38, 3.01)	1.00 (0.28, 3.55)	1.43 (0.50, 4.15)	1.90 (0.49, 7.31)	G
**Nausea or Vomiting**					
A	1.03 (0.58, 2.15)	0.58 (0.21, 1.65)	**1.97 (1.05, 4.05)**		
0.97 (0.47, 1.71)	B	0.56 (0.16, 1.76)	1.91 (0.74, 4.61)		
1.71 (0.61, 4.84)	1.79 (0.57, 6.31)	D	**3.39 (1.04, 12.17)**		
**0.51 (0.25, 0.95)**	0.52 (0.22, 1.35)	**0.29 (0.08, 0.96)**	E		
**Decreased appetite**					
A	1.02 (0.32, 3.24)	0.81 (0.21, 3.16)	**2.30 (1.01, 6.29)**		
0.98 (0.31, 3.16)	B	0.80 (0.13, 4.66)	2.26 (0.54, 11.15)		
1.23 (0.32, 4.78)	1.26 (0.21, 7.45)	D	2.88 (0.59, 15.98)		
**0.43 (0.16, 0.99)**	0.44 (0.09, 1.85)	0.35 (0.06, 1.69)	E		
**Dyspnoea**					
A	1.08 (0.53, 1.96)	0.72 (0.24, 2.34)	2.16 (0.71, 6.87)		
0.93 (0.51, 1.88)	B	0.67 (0.20, 2.66)	2.00 (0.57, 7.91)		
1.39 (0.43, 4.20)	1.48 (0.38, 5.07)	D	2.97 (0.58, 14.21)		
0.46 (0.15, 1.41)	0.50 (0.13, 1.76)	0.34 (0.07, 1.71)	F		

Notes: 95% CI = 95% confidence interval, OR = odds ratio, Odds ratio (95% CI) below the treatments should be read from row to column while above the treatments should be read from column to row. A = Erlotinib + Placebo; B = Erlotinib + Tivantinib; D = Erlotinib + Onartuzumab; E = Erlotinib + Sunitinib; F = Erlotinib + Entinostat; G = Erlotinib + Sorafenib.

### Cumulative ranking probabilities of eight targeted therapies

Cumulative ranking probabilities of eight targeted therapies for advanced/metastatic NSCLC are illustrated in Table 5. The results of SUCRA values demonstrated in the aspect of efficacy, in terms of PFS, the cumulative ranking probability of erlotinib + celecoxib regimen was the highest (83.0%); in terms of OS, erlotinib + tivantinib regimen had the highest cumulative ranking probability (93.3%); in terms of ORR, the cumulative ranking probability of erlotinib + bevacizumab regimen was the highest (86.4%) while that of erlotinib + entinostat regimen was the lowest (23.9%); in terms of DCR, erlotinib + sorafenib regimen had the highest cumulative ranking probability (75.2%). Regarding adverse events, the cumulative ranking probability of erlotinib + onartuzumab regimen was the highest in diarrhea (88.0%), nausea or vomiting (93.0%), decreased appetite (79.8%) and dyspnea (84.5%); the cumulative ranking probability of erlotinib + placebo regimen was the highest in fatigue or asthenia (76.5%); but the cumulative ranking probability of erlotinib + sunitinib regimen was the lowest in diarrhea (21.8%), nausea or vomiting (27.8%) and decreased appetite (29.5%). The cumulative ranking probability of erlotinib + entinostat regimen was the lowest in fatigue or asthenia (30.8%) and dyspnea (32.3%).

**Table 5:** SUCRA values of eight treatment modalities under nine endpoint outcomes

Treatments	SUCRA values (%)								
	PFS (months)	OS (months)	ORR	DCR	Diarrhea	Fatigue or Asthenia	Nausea or Vomiting	Decreased appetite	Dyspnoea
**A**	22.2	36.3	54.1	28.2	67.2	76.5	66.3	71.3	70.5
**B**	73.2	93.3	76.0	73.7	82.3	63.5	62.8	69.5	63.0
**C**	83.0	NR	38.3	51.5	NR	NR	NR	NR	NR
**D**	NR	NR	NR	NR	88.0	69.8	93.0	79.8	84.5
**E**	56.0	63.8	76.9	55.7	21.8	40.2	27.8	29.5	NR
**F**	NR	NR	23.9	NR	50.8	30.8	NR	NR	32.3
**G**	65.6	57.0	44.9	75.2	39.2	69.5	NR	NR	NR
**H**	NR	NR	86.4	65.3	NR	NR	NR	NR	NR

Notes: SUCRA = surface under the cumulative ranking curves; PFS = progression-free survival; OS = overall survival; ORR = overall response rate; DCR = disease control rate; NR = not report; A = Erlotinib + Placebo; B = Erlotinib + Tivantinib; C = Erlotinib + Celecoxib; D = Erlotinib + Onartuzumab; E = Erlotinib + Sunitinib; F = Erlotinib + Entinostat; G = Erlotinib + Sorafenib; H = Erlotinib + Bevacizumab.

**Figure 4:**
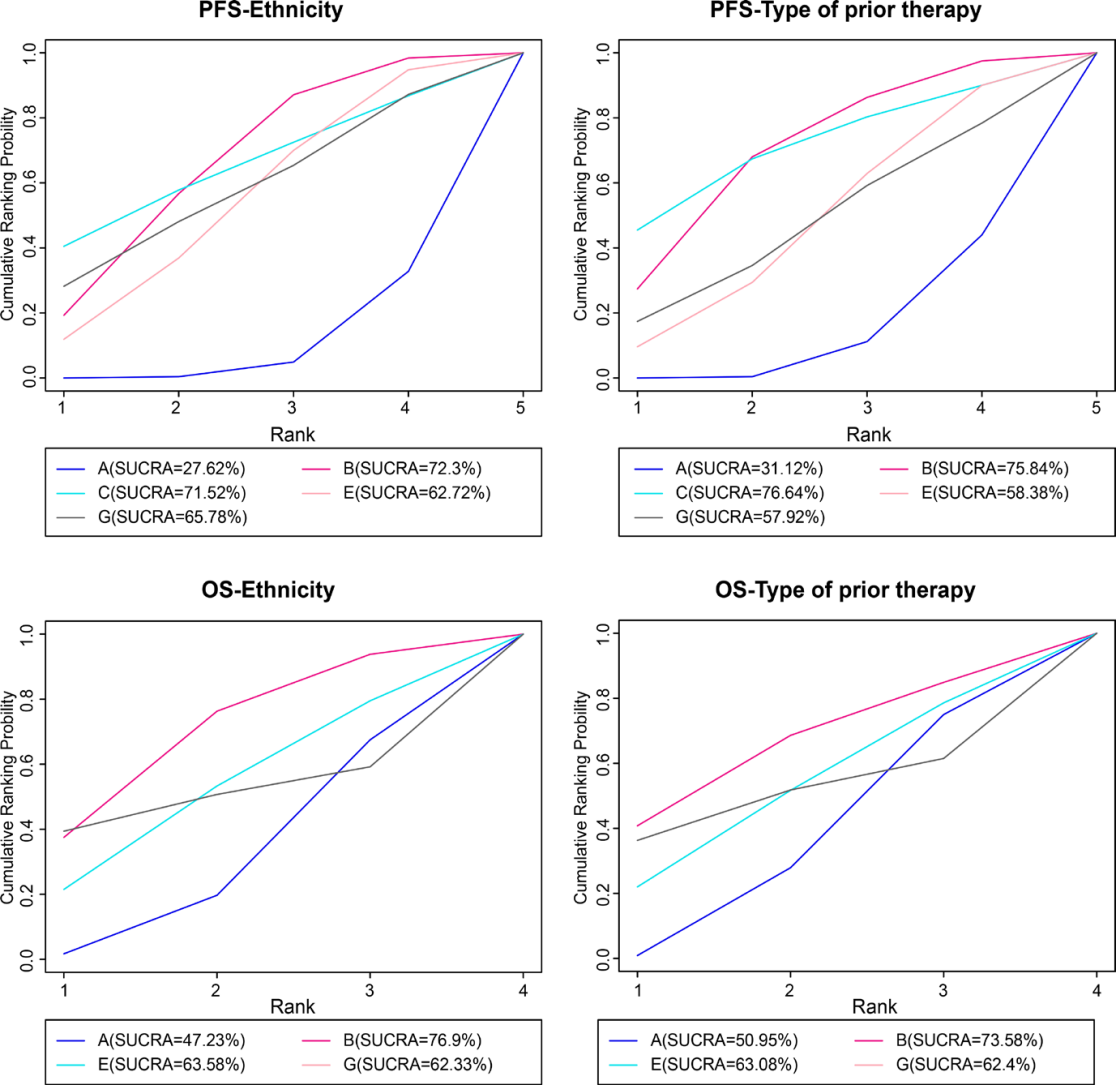
The cumulative sorting probability map of PFS and OS by meta regression analysis after correction Note: PFS = progression-free survival; OS = overall survival; A = erlotinib + placebo regimen; B = erlotinib + tivantinib regimen; C = erlotinib + celecoxib regimen; E = erlotinib + sunitinib regimen; G = erlotinib + sorafenib regimen.

Moreover, the information about ethnicity and prior therapy was included according to PFS and OS indicators, and meta regression analysis was performed for revision of PFS and OS results. SUCRA curves were drawn to re-sequenced interventions. In terms of PFS-Ethnicity, the result of intervention after correction showed a minor deviation compared with that before correction. Erlotinib + celecoxib regimen before correction showed the highest value of cumulative sort probability, while showed the second after correction (71.52%), which was lower than erlotinib + tivantinib regimen (72.3%). Therefore, it indicated the effect of ethnicity on the patient’s survival. However, for PFS-Type of prior therapy, OS-Ethnicity and OS-Type of prior therapy, the result of interventions after correction were in line with that before correction, indicating no obvious heterogeneity. In conclusion, the results of our study are reliable as the heterogeneity was controlled by meta-analysis, which showed important significance for targeted therapies in treatment with advanced/ metastatic NSCLC patients (Figure 4).

## DISCUSSION

The pairwise meta-analysis suggested that erlotinib + tivantinib, and erlotinib + celecoxib regimens had better long-term efficacy for the treatment of advanced/metastatic NSCLC. However, the adverse events of diarrhea, nausea or vomiting, decreased appetite, and dyspnea, exhibited the highest SUCRA value in erlotinib + onartuzumab regimen. For diarrhea, nausea or vomiting, and decreased appetite, the erlotinib + sunitinib regimen exhibited the lowest SUCRA; for fatigue or asthenia, and dyspnea, the erlotinib + entinostat regimen exhibited the lowest SUCRA value. The tyrosine kinase c-MET acts as a tumor suppressor in NSCLC, and as a resistance mediator to erlotinib in EGFR-activating mutations [10]. When erlotinib is given with tivantinib, which is a novel, selective inhibitor of c-MET, to patients with advanced/metastatic NSCLC, tivantinib can contribute to prolonged PFS and improved OS [5]. Moreover, erlotinib and afatinib could be the best choice for patients with chemo-naïve EGFR mutations. In addition, erlotinib has a potential survival benefit in patients who have previously been treated [18]. Scagliotti *et al.* reported that erlotinib plus sunitinib exhibited antitumor activities by inhibiting tumor growth, metastasis, and angiogenesis, and were associated with a longer PFS and greater ORR [7]. However, a previous study showed that treatment-related adverse events from the combination of erlotinib and sunitinib were more frequent than from erlotinib alone, including diarrhea, anorexia, fatigue, nausea, and dyspnea; this is consistent with our results [4]. Combination of bevacizumab and erlotinib has been effective in prolonging the PFS and ORR in patients, while having minimal side effects [1]. Targeting multiple molecular pathways can increase the efficacy and avoid resistance development, without increasing adverse events (AEs) [19]. A phase II study of erlotinib, placebo-controlled and randomized, without and with entinostat, was conducted for treatment of patients with advanced NSCLC [12].

Based on our results and SUCRA values, erlotinib + tivantinib, and erlotinib + onartuzumab regimens had fewer adverse events, while erlotinib + sunitinib had a higher incidence of adverse events for patients with advanced/metastatic NSCLC. Onartuzumab can bind the c-MET extracellular domain to inhibit hepatocyte growth factor binding and activation, thus contributing to the improved PFS and OS in patients with advanced/metastatic NSCLC [10]. As for the safety, erlotinib + sunitinib, and erlotinib + entinostat regimens ranked lower, indicating that these two targeted therapies might have more side effects. Previous studies have indicated that entinostat may inhibit epigenetic modifications to reverse the resistance to epidermal growth factor receptor tyrosine kinase inhibitor therapy in advanced/metastatic NSCLC [20, 21]. However, erlotinib + onartuzumab with the highest cumulative ranking probabilities indicated a relatively lower incidence of adverse events, which is consistent with our results of network meta-analysis.

Our present study systematically compared the efficacy and adverse events of eight erlotinib-based targeted therapies for advanced/metastatic NSCLC with direct and indirect evidence [22]. However, this network meta-analysis has some limitations. First, the number of randomized controlled trials included in this study was relatively small, which could have an influence on the universality of our results. Second, information of some outcome indicators was not complete; therefore, we did not use the node-splitting method to analyze the inconsistency of outcome indicators and also did not carry out a cluster analysis of the SUCRA values.

There was no statistical difference in short-term efficacy, while erlotinib + tivantinib, and erlotinib + celecoxib regimens had a better long-term efficacy in patients with advanced/metastatic NSCLC. Regarding the adverse events, they were higher for erlotinib + sunitinib, and erlotinib + entinostat regimens; this may provide clinical guidelines for targeted therapies for patients with advanced/metastatic NSCLC. However, the validity of our results may be affected by the fact that seven out of ten included studies did not analyze the c-Met levels. In addition, our results suggest that ethnicity has a significant influence on patient PFS. Future studies should analyze the effect of ethnicity on the NSCLC patient survival, as well as include more RCTs.

## MATERIALS AND METHODS

### Retrieval strategy

The electronic retrieval of English databases, PubMed and Cochrane Library, was conducted from their inception to February 2017, supplemented with manual retrieval of relevant references. The electronic retrieval combined keywords and free words to search for references. The keywords included advanced or metastatic NSCLC, targeted therapies, and randomized controlled trials.

### Inclusion and exclusion criteria

Inclusion criteria were as follows: (1) study type: randomized controlled trial; (2) treatment regimen: erlotinib + placebo, erlotinib + tivantinib, erlotinib + celecoxib, erlotinib + onartuzumab, erlotinib + sunitinib, erlotinib + entinostat, erlotinib + sorafenib and erlotinib + bevacizumab, all regimens were second-line or beyond; (3) study subject: patients with advanced/metastatic NSCLC, aged 20–90 years old, receiving radiotherapy, chemotherapy or radiochemotherapy prior to research subject recruitment; (4) outcome indicator: progression-free survival (PFS), overall survival (OS), overall response rate (ORR), disease control rate (DCR), diarrhea, fatigue or asthenia, anemia, nausea or vomiting, decreased appetite, dyspnea and neutropenia. Exclusion criteria were as follows: (1) patients with brain metastases or spinal cord compression; (2) patients without adequate blood or liver/kidney function; (3) patients with uncontrolled hypertension in the past 12 months or clinically significant cardiovascular disease; (4) non-randomized control trial; (5) letters, reviews or summaries; (6) non-English studies or non-human studies; (7) studies without complete data (e.g. not paired study); (8) duplicate studies.

### Data extraction and quality assessment

The data contained in the included studies were collected by two researchers independently with the application of unified data collection form. Disagreements between two reviewers were resolved by discussion with other researchers until consensus was achieved. Two or more researchers assessed included studies with the Cochrane risk of bias assessment tool [23]. The assessment included assigning a judgment of “yes,” “no,” or “unclear” for each domain to designate a low, high, or unclear risk of bias, respectively. A study with no more than 1 “unclear” or “no” domain would be identified as having a low risk of bias; a study with 2–3 “unclear” or “no” domains would be regarded as having an unclear risk of bias; and a study with over 4 “unclear” or “no” domains would be deemed as having a high risk of bias [24]. Review Manager 5 (RevMan 5.2.3, Cochrane Collaboration, Oxford, UK) was used to assess quality assessment and investigate publication bias.

### Statistical analysis

Initially, traditional pairwise meta-analyses were performed to compare the eight targeted therapies. The pooled estimates of weighted mean differences (WMD), odds ratios (OR) and 95% confidence intervals (CIs) were calculated. The heterogeneity test of the studies was performed by Chi-square test and I-square test [25]. The R3.2.1 software was used to draw network diagrams of the targeted therapies. Every node represents one targeted therapy; the node size represents the number of the corresponding targeted therapy; the line thickness between two nodes represents the number of paired studies of two targeted therapies. In addition, we carried out Bayesian network meta-analyses to compare eight targeted therapies to each other as well. Each analysis was based on non-informative priors for effect sizes and precision. Convergence and lack of auto correlation were checked and confirmed after four chains and a 20,000-simulation burn-in phase. Finally, direct probability statements were derived from an additional 50,000-simulation phase [26]. To assist in the interpretation of WMDs or ORs, we calculated the probability of each targeted therapy being the most effective or safest treatment method which was based on a Bayesian approach using probability values summarized as the surface under the cumulative ranking curve (SUCRA). A targeted therapy with a larger SUCRA value represents a better efficacy [27, 28]. All calculations were done using R (V.3.2.1) package (V.0.6) as well as the Markov Chain Monte Carlo engine Open BUGS (V.3.4.0).

## SUPPLEMENTARY MATERIALS FIGURES AND TABLE




