# Supplementary material for: Meta-analysis of efficacy and adverse events of erlotinib-based targeted therapies for advanced/metastatic non-small cell lung cancer
Source: Oncotarget. 2017 Jul 31;8(49):86816–27. doi: 10.18632/oncotarget.19735 (PMC5689727; doi:10.18632/oncotarget.19735)
Supplement: Supplementary file 1 [file oncotarget-08-86816-s001.pdf]

Meta-analysis of efficacy and adverse events of erlotinib-based targeted therapies for advanced/metastatic non-small cell lung cancer

SUPPLEMENTARY MATERIALS

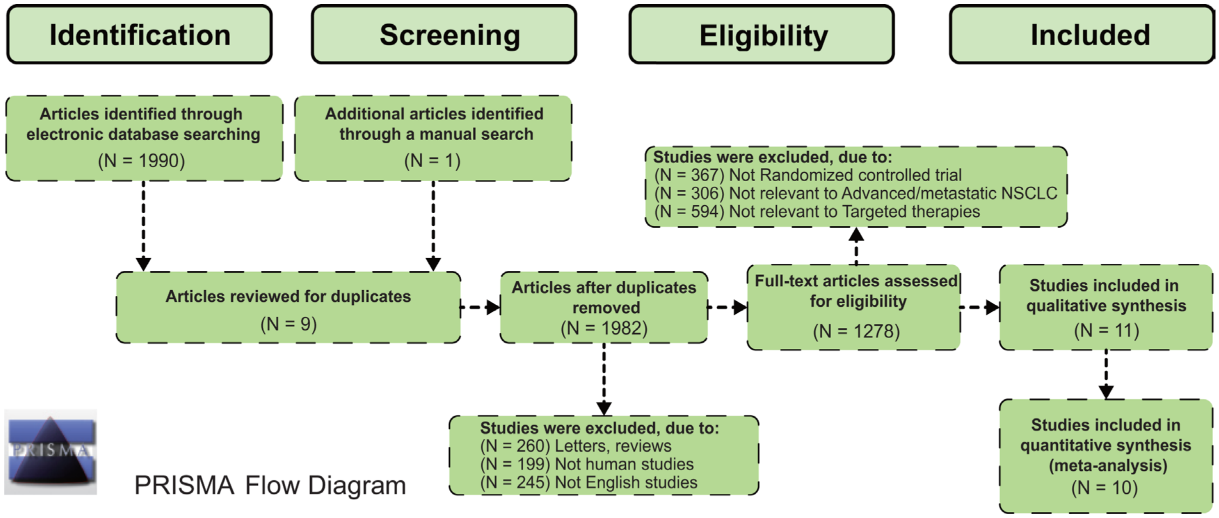

Supplementary Figure 1: Flow diagram of the literature search.

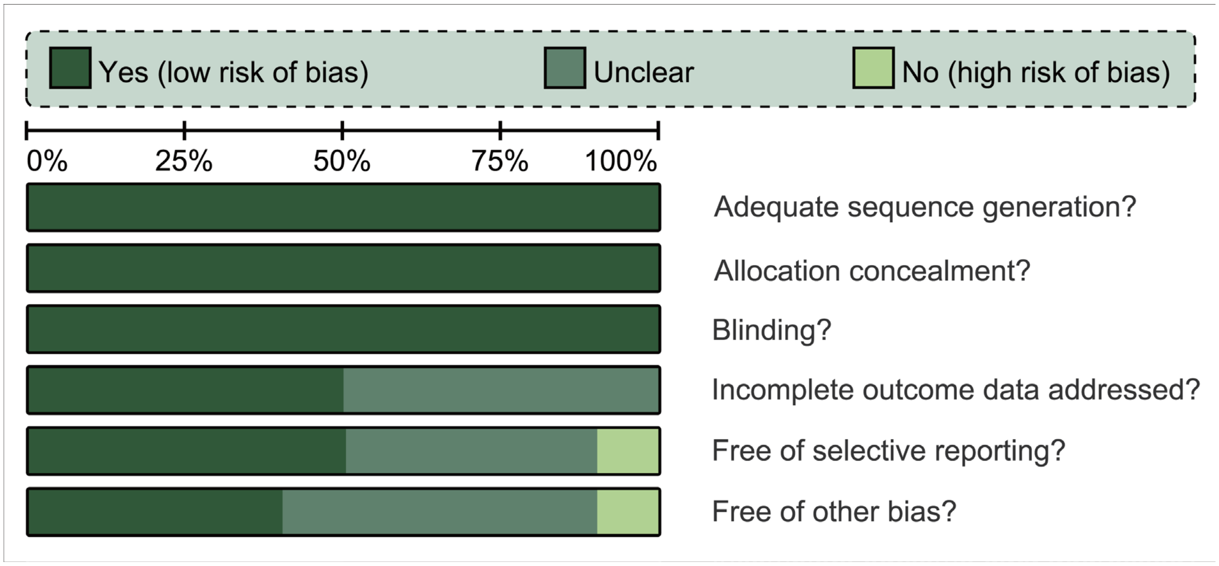

Supplementary Figure 2: Cochrane risk of bias graph of included studies.

Supplementary Table 1: The baseline characteristics for included studies. See Supplementary_Table_1
