# Supplementary material for: Meta-analysis of efficacy and adverse events of erlotinib-based targeted therapies for advanced/metastatic non-small cell lung cancer
Source: Oncotarget. 2017 Jul 31;8(49):86816–27. doi: 10.18632/oncotarget.19735 (PMC5689727; doi:10.18632/oncotarget.19735)
Supplement: Supplementary file 2 [file oncotarget-08-86816-s002.docx]

**Supplementary Table 1: The baseline characteristics for included studies.**

| First author | Year | Country | Ethnicity | Follow-up | Type of prior therapy | Interventions | | Total | Sample size | | EGFR wild type (%) | | MET status (overexpressed) (%) | | Gender (M/F) | | Age (years) | |
| --- | --- | --- | --- | --- | --- | --- | --- | --- | --- | --- | --- | --- | --- | --- | --- | --- | --- | --- |
|  |  |  |  |  |  | T1 | T2 |  | T1 | T2 | T1 | T2 | T1 | T2 | T2 | T2 | T1 | T2 |
| Yoshioka H | 2015 | Japan | Asians | 2Y | CT (platinum-based) | A | B | 307 | 153 | 154 | 100% | 100% | 50.33% | 53.90% | 102/51 | 109/45 | 63(27–84) | 63(33–83) |
| Scagliotti G | 2015 | Italy | mixed | 2Y | CT (platinum-based) | A | B | 1048 | 522 | 526 | 89.66% | 89.16% | 19.92% | 20.34% | 309/213 | 310/216 | 61.1 ± 9.8 | 61.2 ± 10.1 |
| Reckamp KL | 2015 | America | mixed | 1Y | CT(NR) | A | C | 107 | 53 | 54 | 50.94% | 57.41% | NR | NR | 24/29 | 26/28 | 65(30-80) | 63.5(41-80) |
| Spigel DR | 2013 | America | mixed | 2Y | CT (platinum-based) | A | D | 137 | 68 | 69 | 73.53% | 71.01% | 45.59% | 50.72% | 42/26 | 40/29 | 63(42-83) | 64(30-83) |
| Groen HJ | 2013 | America | mixed | 1.5Y | CT (platinum-based)/RT | A | E | 132 | 67 | 65 | NR | NR | NR | NR | 45/22 | 39/26 | 61(39-81) | 59(37-79) |
| Witta SE | 2012 | America | mixed | 2.5Y | CT(NR)/RT | A | F | 132 | 65 | 67 | NR | NR | NR | NR | 43/22 | 39/28 | NR | NR |
| Scagliotti GV | 2012 | Italy | mixed | 2Y | CT (platinum-based)/bev | A | E | 960 | 480 | 480 | NR | NR | NR | NR | 284/196 | 297/183 | 61(30-82) | 61(31-85) |
| Spigel DR | 2011 | America | Caucasians | 1.5Y | CT(NR)/bev | A | G | 166 | 55 | 111 | 88.89% | 95.56% | NR | NR | 26/29 | 62/49 | 65(45-83) | 65(44-88) |
| Sequist LV | 2011 | America | Caucasians | 1.5Y | CT(NR) | A | B | 167 | 83 | 84 | 57.83% | 60.71% | NR | NR | 49/34 | 51/33 | 62(23-89) | 64(32-81) |
| Herbst RS | 2011 | America | mixed | 3Y | CT(NR)/RT | A | H | 636 | 317 | 319 | 89.41% | 93.51% | NR | NR | 170/147 | 171/148 | 65 ± 10.3 | 64.8 ± 10.4 |

Notes: T = treatment; M = male; F = female; Y = years; mixed = mixed population; CT = chemotherapy; RT = radiation therapy; CRT = chemoradiotherapy; CT (NR) = chemotherapy, but not reported whether based on platinum or not; NR = not report; bev = bevacizumab; A = Erlotinib + Placebo; B = Erlotinib + Tivantinib; C = Erlotinib + Celecoxib; D = Erlotinib + Onartuzumab; E = Erlotinib + Sunitinib; F = Erlotinib + Entinostat; G = Erlotinib + Sorafenib; H = Erlotinib + Bevacizumab.
